# Effects of autologous bone marrow stem cell transplantation on beta-adrenoceptor density and electrical activation pattern in a rabbit model of non-ischemic heart failure

**DOI:** 10.1186/1749-8090-1-17

**Published:** 2006-06-26

**Authors:** Stefan Dhein, Jens Garbade, Djazia Rouabah, Getu Abraham, Fritz-Rupert Ungemach, Katja Schneider, Cris Ullmann, Heike Aupperle, Jan Fritz Gummert, Friedrich-Wilhelm Mohr

**Affiliations:** 1 Clinic for Cardiac Surgery, Heart Centre Leipzig, University of Leipzig, Germany; 2 Institute for Pharmacology, Pharmacy and Toxicology, Faculty of Veterinary Medicine, University of Leipzig, Germany; 3 Institute of Veterinary Pathology, University of Leipzig, Faculty of Veterinary Medicine, Germany

## Abstract

**Background:**

Since only little is known on stem cell therapy in non-ischemic heart failure we wanted to know whether a long-term improvement of cardiac function in non-ischemic heart failure can be achieved by stem cell transplantation.

**Methods:**

White male New Zealand rabbits were treated with doxorubicine (3 mg/kg/week; 6 weeks) to induce dilative non-ischemic cardiomyopathy. Thereafter, we obtained autologous bone marrow stem cells (BMSC) and injected 1.5–2.0 Mio cells in 1 ml medium by infiltrating the myocardium via a left anterolateral thoracotomy in comparison to sham-operated rabbits. 4 weeks later intracardiac contractility was determined in-vivo using a Millar catheter. Thereafter, the heart was excised and processed for radioligand binding assays to detect β_1_- and β_2_-adrenoceptor density. In addition, catecholamine plasma levels were determined via HPLC. In a subgroup we investigated cardiac electrophysiology by use of 256 channel mapping.

**Results:**

In doxorubicine-treated animals β-adrenoceptor density was significantly down-regulated in left ventricle and septum, but not in right ventricle, thereby indicating a typical left ventricular heart failure. Sham-operated rabbits exhibited the same down-regulation. In contrast, BMSC transplantation led to significantly less β-adrenoceptor down-regulation in septum and left ventricle. Cardiac contractility was significantly decreased in heart failure and sham-operated rabbits, but was significantly higher in BMSC-transplanted hearts. Norepinephrine and epinephrine plasma levels were enhanced in heart failure and sham-operated animals, while these were not different from normal in BMSC-transplanted animals. Electrophysiological mapping revealed unaltered electrophysiology and did not show signs of arrhythmogeneity.

**Conclusion:**

BMSC transplantation improves sympathoadrenal dysregualtion in non-ischemic heart failure.

## Background

Stem cell transplantation, in particular transplantation of mesenchymal autologous bone marrow stem cells, is currently discussed to be a possible alternative to heart transplantation and may be of therapeutic interest in ischemic heart disease [1-3]. The use of autologous bone marrow stem cells (BMSC) has the advantage of non-immunogeneity. Until now there are a number of experimental [4-6] and also clinical [7-11] studies demonstrating positive effects of stem cell therapy in ischemic heart disease. However, at present there is only little knowledge on the use of autologous stem cells in non-ischemic cardiomyopathy. Thus, we decided to investigate the effect of autologous bone marrow stem cell transplantation in doxorubicine-induced non-ischemic cardiomyopathy in rabbits using direct epicardial infiltration for delivery.

A special problem with the evaluation of such a therapy is to get an impression of the long-term improvement in hemodynamics and in the sympathoadrenal regulation. Thus, echocardiography and related methods give a measure for the acute contractility at the moment of investigation, but do not allow an insight into the sympathoadrenal dysregulation typical for heart failure [12] or its possible normalisation by therapy. The idea of our study was to assess the effect of BMSC transplantation therapy on non-ischemic heart failure by measurement of contractility and β-adrenoceptor density using (-)-^125 ^[I ]-iodocyanopindolol radioligand binding assay: it is long known that heart failure leads to compensatory activation of sympathoadrenal system with enhanced release of catecholamines and to down-regulation of cardiac β_1_-adrenoceptors [13,14], which is correlated to the severity of the disease [15,16], affecting mostly β_1_-adrenoceptors while β_2_-adrenoceptors remained unaltered in most studies [17] and finally resulting in a decrease in β-adrenoceptor density. Remaining β-adrenoceptors are desensitized, probably mostly via G-protein coupled receptor kinases (GRK2 and/or GRK5) [17-22] and to some extent via pathways involving PKA [23]. The down-regulation of β-adrenoceptor number as assessed via radioligand binding studies mirrors the severity of chronic heart failure [14]. This holds also true for rabbits suffering from chronic heart failure [24].

Treatment of chronic heart failure with the β-blocker metoprolol results in hemodynamic improvement and up-regulation of β-adrenoceptor density [25,26]. The process of β-adrenoceptor down-regulation in chronic heart failure takes days to weeks and the extent of this reduction in β-adrenoceptor number is directly related to the severity of heart failure [13,14,27], thus, reflecting the long-term hemodynamic situation. Inversely, an effective therapy of heart failure should lead to better contractility and in consequence a reduction of heart failure-induced enhancement in catecholamine levels and finally a (partial) normalisation of β-adrenoceptor density. Therefore, we decided to measure β-adrenoceptor density, contractility and catecholamine levels in normal rabbits, doxorubicine-induced non-ischemic cardiomyopathy with or without autologous BMSC transplantation or sham-operation.

## Methods

The study was performed in accordance to the German laws for animal health and protection declaration and was approved by the local authorities, Government of Leipzig (Reg.-Nr.: 24-9168.11-11/03). We used male White New Zealand rabbits of 1500–2000 g body weight (at beginning of the study) (conventional, normally fed ad libitum) (Charles River, Kisslegg, Germany).

### Animal model and investigation of contractility

Heart failure was induced by repeated i.v. injection of doxorubicine 3 mg/kg/week over 6 weeks, followed by a 14 days doxorubicine-free interval. Animals that survived 2 weeks after heart failure induction were randomized to either no treatment, sham operation or BMSC transplantation. After intramuscular anaesthesia with ketamin (50 mg/kg) and xylazine (5 mg/kg), we obtained autologous mesenchymal bone marrow stem cells from a femur punction, isolated the mononuclear stem cell fraction, and after subcultivation for 4 days (see below), the stem cells were injected directly into the wall of the left ventricle. For transplantation rabbits were anaestetized (0.8 vol% isofluran, protocol see [28]. Left thoracotomy through the intercostal space was performed and the left ventricle was exposed. Either BMSC (1.5 – 2.0 Mio cells in 1 ml) or medium (1 ml) was injected at 4 positions into the tissue of the free lateral wall of the left ventricle in a circular manner with a 1 ml syringe within 2 minutes.

28 days after the operation, animals were subjected to the final experiment. One hour prior to surgery (anaesthesia as described above), we collected 2 ml venous blood in potassium EDTA monovettes (with addition of 1 μM glutathione/ml plasma) from the rabbits after at least 30 min at rest for determination of plasma catecholamines. After centrifugation (600 g/10 min/4°C), plasma was removed, quickly frozen in liquid nitrogen, and stored at -80°C until further use. We introduced a Millar catheter designed for rabbits (FMI, Germany) via the femoral artery and registered pressure-volume loops under the influence of 1.5 μg/kg dopamine, in order to assess the contractile function. dP/dt_max _and dP/dt_min _were determined from these measurements. Subsequently, we excised the heart for mapping experiments and radioligand binding assay. After the experiment, tissue was immediately placed in cardioplegic solution. Left ventricle, septum and right ventricle were excised and subsequently shock-frozen.

We investigated 4 groups of rabbits: healthy control animals (n = 10), doxorubicine-induced heart failure (n = 6), doxorubicine-induced heart failure with sham operation (n = 8), and doxorubicine-induced heart failure with stem cell treatment (n = 14). During the investigations and subsequent evaluations the experimentators were blinded.

### Stem cell purification and subcultivation

Under sterile conditions we obtained 1.5 – 2 ml bone marrow in 2 mM EDTA containing phosphate buffered saline (PBS) from a femur punction. The mixture was initially centrifuged at 300 g, at room temperature (RT), for 5 min, and the resulting pellet was resuspended in PBS containing 2 mM EDTA, and separated in a Ficoll (1.073 g/ml) density gradient centrifugation (cell suspension : ficoll: 1.5: 1.0; 30 min centrifugation at 500 g, RT). The mononuclear fraction interphase was collected, washed twice in PBS/EDTA. The final pellet was resuspended in 12 ml Dulbeccos Modefied Eagle Medium (DMEM) cell culture medium (+ 10% fetal calf serum, 100 U/ml penicillin, 100 μg/ml streptomycine; 37°C, 5% CO_2_) and seeded on gelatine-coated petridishes. For the first 24 hours cells were incubated with additional 10 μM 5-azacytidine. Cells were cultured for 4 days. Cell culture medium was changed every 48 h, and non-adherent hematopoetic cells were discarded. Prior to transplantation cells were detached with trypsin/EDTA, and incubated with 2.5 μM Vybrand DiI cell labelling solution (Molecular Probes) for 0.5 h protected from light in the incubator at 37°C followed by 3 washing steps in PBS. Finally, the resulting mesenchymal mononuclear c-kit positive bone marrow stem cells (tested using a commercial anti-c-kit antibody) were suspended in DMEM for injection (1.5–2.0 Mio. cells in 1 ml).

### Radioligand binding study

β-adrenoceptors were assessed by (-) [^125^I]-iodocyanopindolol (ICYP)-binding assay described elsewhere [29]. Briefly, tissue samples from right ventricle, left ventricle and septum were homogenised in 10 volumes of ice-cold 1 mmol/l KHCO_3 _with an Ultra Turrax (Janke and Kunkel, Staufen, Germany), diluted to 20 ml with 1 mmol/l KHCO_3_, centrifuged at 500 g for 10 min, passed through 4 layers of cheesecloth, and centrifuged again at 50,000 g for 20 min. Pellets were washed once by resuspension and recentrifugation and finally resuspended in incubation buffer (Tris-HCl 10, NaCl 154, ascorbic acid 0.55 mmol/l, pH 7.4, 25°C) at a protein concentration of 0.1–0.2 mg/ml. Protein content was determined by the method of Lowry using bovine serum albumine as a standard.

The density of β-adrenoceptors in cardiac membranes was determined by (-) [^125^I]ICYP binding at six concentrations ranging from 5 to 200 pmol/l as detailed elsewhere [29]. Non-specific binding of ICYP was defined as binding to membranes which could not be displaced by a high concentration of the non-selective β-adrenoceptor antagonist (±) CGP 12177 (1 μmol/l). Specific binding was defined as total binding minus non-specific binding and usually was about 70–80% at 50 pmol/l ICYP.

To determine the relative amounts of β_1_- and β_2_-adrenoceptors, membranes were incubated with increasing concentrations of the β2-adrenoceptor antagonist ICI 118,551 (10^-10 ^– 10^-4 ^M) and a constant concentration of ICYP (100 pmol/l), and the specific binding assessed as described above (using CGP12177). Binding curves were analysed using the iterative curve fitting program GraphPadPrism (GraphPad Software, San Diego, CA, USA).

### High Pressure Lquid Chromatography (HPLC)

Plasma norepinephrine content was assessed by high-pressure liquid chromatography (HPLC) and electrochemical detection [30] using a commercial HPLC assay (Chromsystems, Martinsried, Germany) based on the protocol by Goldstein [31] and by Hjemdahl et al. [32]. Briefly, catecholamines were adsorbed to aluminiumoxide (in prepacked columns; Chromsystems, Martinsried, Germany) by shaking 1 ml plasma with 0.5 ml extraction buffer (Tris-buffer) and aluminiumoxide for 10 min. As internal standard 600 pg dihydroxybenzylamine were added to the plasma sample. After 10 min, the probe was washed twice and washing buffer was removed. Thereafter, the bound catecholamines and internal standard were eluted using 120 μl elution buffer (Chromsystems, Martinsried, Germany) and filtration (at 700 g). 40 μl probe volume were injected at a flow of 1 ml/min (HPLC autosampler (GINA50) and pump (P580): Gynkotec, Germering, Germany; mobile phase 5001; Chromsystems, Martinsried, Germany) on a pre-equilibrated RP18 column (Chromsystems, Martinsried, Germany). The HPLC system was controlled by the Chromeleon software 4.10 (Gynkotec, Germering, Germany). Catecholamines were detected using an electrochemical detector (DECADE; flow cell: VT-03; Fa. ANTEC Comp., Leyden, Netherlands) (a working potential of 0.55 V yielded maximum signals with lowest noise). An external calibration standard containing 5 ng/ml norepinephrine, 5 ng/ml dihydroxybenzylamine, 2.5 ng/ml epinephrine and 2.5 ng/ml dopamine was also used for each experiment. Each sample was injected three times and the concentration was determined as the mean of these three detections.

### Histology

Tissue samples of the hearts were embedded in paraffin and 5 μm sections of the hearts were stained with picrosirius red according to standard protocols. Using fluorescence microscopy we identified the BMSC or their remnants by Vybrand-DiI-red fluorescence and counted the number of these BMSC (or remains) per visual field at 200 x magnification. Moreover, we looked for signs of inflammation, i.e. leukocyte/lymphocyte infiltration.

### Mapping experiments

In order to investigate, whether BMSC injection might produce inhomogeneities in the cardiac electrical activation pattern or autonomic areas, we submitted 4 hearts of each group to epicardial mapping as previously described [33]. Briefly, rabbits were anaesthetized by isoflurane, the heart was excised and prepared according to the Langendorff technique (constant pressure: 70 cm H2O, perfusion with Tyrode solution (Na^+ ^161.02, K^+ ^5.36, Ca^++ ^1.8, Mg^++ ^1.05, Cl^- ^147.86, HCO3^- ^23.8, PO4^2- ^0.42 and glucose 11.1 mM, equilibrated with 95% O2 and 5% CO2 (pH = 7.4); the surface temperature of the heart was 37°C). The hearts, which were beating at their spontaneous rate, were connected to a 256 channel mapping system HAL4 (Ing. Buero Peter Rutten, Hamburg, Germany, temporal resolution: 20 kHz per channel; amplitude resolution: 0.04 mV, interchannel coupling <-60 decibel; bandwidth of the system: 0.5 Hz – 100 kHz, data were not filtered) as described previously [33]. 256 AgCl electrodes (1 mm interelectrodes distance), which were attached to the heart surface in an elastic manner, so that they could follow the heart movements easily without dislocation. The four plates were located at (a) the right wall (64 channels) (b) the front wall (64 channels) (c) the left wall (64 channels) and (d) the back wall (64 channels), so that both ventricles were mapped with a total of 256 electrodes. Activation and repolarization time points at each electrode were determined as t(dU/dtmin) or t(dU/dt_max_), respectively [33-35]. After automatic determination activation and repolarization timepoints were verified (or corrected if necessary) manually by the experimentator. Total activation time (TAT, [ms]) was calculated as the delay between activation of the first and activation of the last electrode. Standard deviation of activation times (SD(ACT)) was used as a measure for local inhomogeneity. For each electrode an activation-recovery-interval (ARI, reflecting epicardial potential duration) was calculated. Inhomogeneity of ARI was analyzed calculating standard deviation of ARI at 256 electrodes (=ARI-dispersion).

### Statistics

All continuous variables are presented as means ± S.E.M. of n experiments. Experimental data to β-adrenoceptors were fitted and analysed by computer-supported iterative non-linear regression analysis using the GraphPadPrism program (GraphPAD Software; San Diego, CA, USA). Statistical significance of differences was analysed by unpaired two-tailed Student's t-test or, if appropriate, by repeated measures ANOVA followed by t-test using Bonferroni corrections for multiple comparisons. P < 0.05 was considered to indicate a significant difference. The statistical evaluation was performed using SYSTAT program (Systat Inc., Evanston, IL, USA).

### Chemicals

CGP12177, ICI118,551 were from Sigma (Taufkirchen, Germany), (-) [^125^I]iodocyanopindolol (ICYP, specific activity: 2200 Ci/mmol) (Perkin Elmer, Boston, MA, USA), all other chemicals were of the purest commercially available grade and obtained from Sigma.

## Results

**Figure 1:**
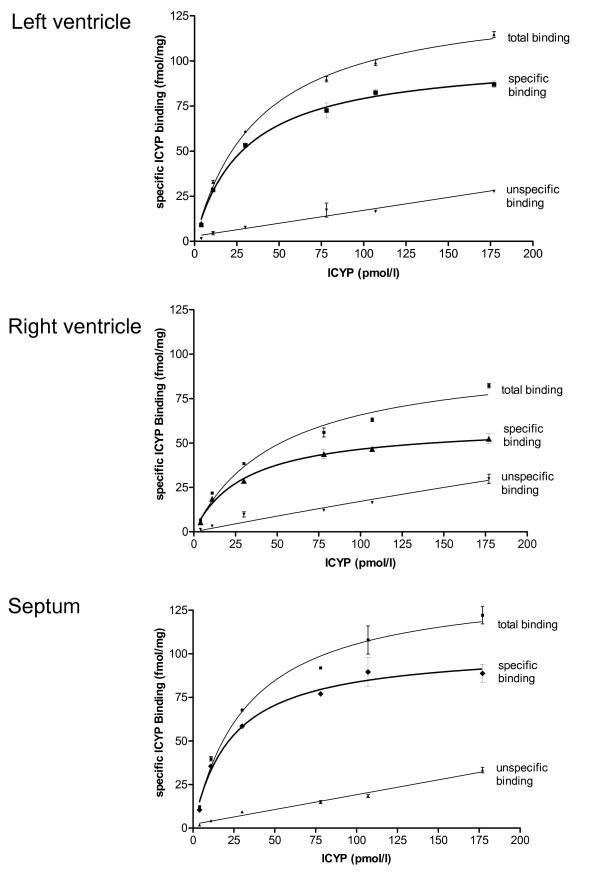
representative original ICYP binding data for one animal (k042) from the control group given for left ventricle, septum and right ventricle as total binding, unspecific binding and specific binding.

**Figure 2:**
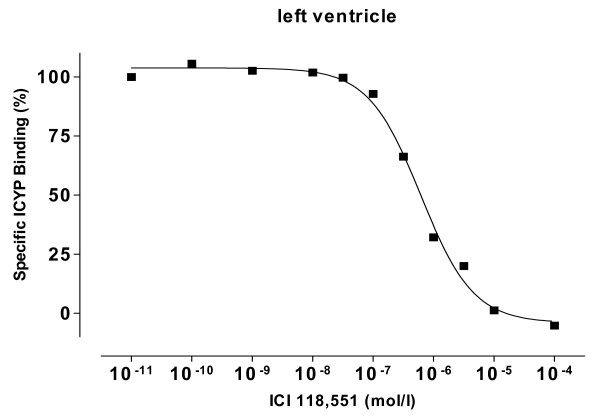
Displacement of ICYP binding by increasing concentrations of the β_2_-adrenoceptor ligand ICI 118, 551, revealing a monophasic competition, which means that there is almost no β_2_-adrenoceptor fraction.

We found saturable ICYP binding in the membrane preparations of all hearts. Unspecific binding was below 20% of total binding at K_D _(example is given in fig. 1, upper panel). Specific binding revealed different β-adrenoceptor densities in left ventricle, septum and right ventricle with lowest values in the right ventricle (fig. 1). Displacement of ICYP by ICI 118,551 revealed a monophasic competition curve and a nearly undetectable β_2_-adrenoceptor population (fig. 2).

**Figure 3:**
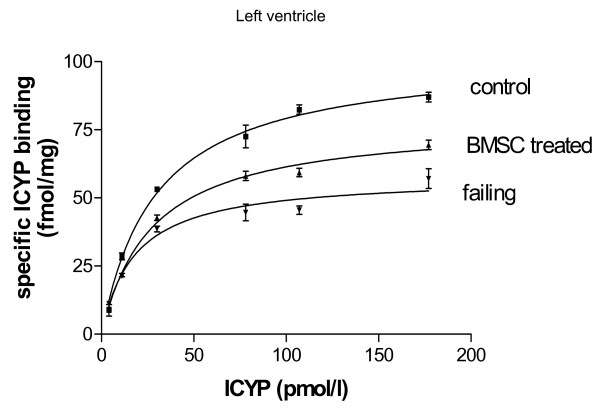
Binding curves for control hearts, failing hearts and BMSC-treated failing hearts showing specific ICYP binding for left ventricle.

**Figure 4:**
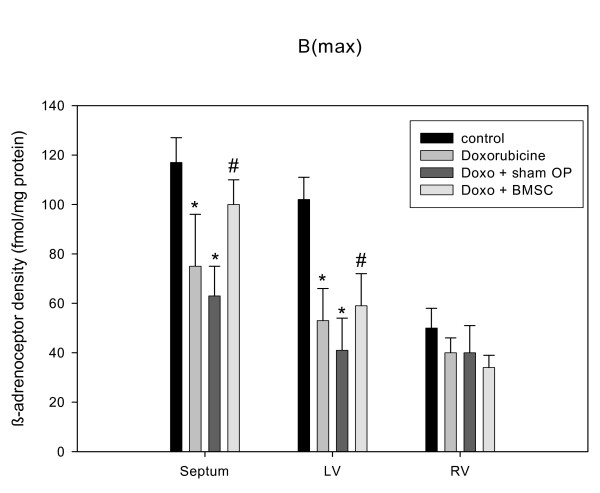
Quantitative data for β-adrenoceptor density in normal hearts, failing hearts, sham-operated failing hearts and BMSC transplanted failing hearts, given for left ventricle, septum and right ventricle as MEANS ± SEM of n = 6 hearts in each group. Significant differences versus normal hearts are indicated by a * (p < 0.05), while significant changes versus sham-operated hearts are indicated by a # (p < 0.05). Corresponding data for KD (septum, left ventricle (LV), right ventricle (RV)) were: 37 ± 3, 35 ± 4, 42 ± 3 (controls), 48 ± 6, 44 ± 6, 48 ± 7 (doxorubicine), 47 ± 5, 46 ± 6, 48 ± 9 (sham operated hearts), 42 ± 9, 53 ± 8, 57 ± 14 (BMSC transplanted hearts); there were no significant differences between the KD values.

Regarding the effects of disease and treatment, we found significant down-regulation of β-adrenoceptor density in untreated heart failure as compared to control (fig. 3). This down-regulation was attenuated in BMSC-treated animals (fig. 3). In sham operated animals with vehicle injection, however, we found nearly the same down-regulation as in failing hearts (fig. 4). The β-adrenoceptor down-regulation in failing hearts was accentuated in septum and left ventricle (fig. 4). Most interestingly, in failing hearts receiving BMSC transplantation the β-adrenoceptor down-regulation was significantly attenuated in septum and left ventricle as compared to sham operated failing hearts (fig. 4). β-adrenoceptor density was nearly normalized in septum of BMSC treated hearts and significantly enhanced in left ventricle (as compared to sham operated hearts), although still below the normal level (fig. 4). K_D _was similar among all groups indicating unchanged affinity (given in the legend to fig. 4).

**Figure 5:**
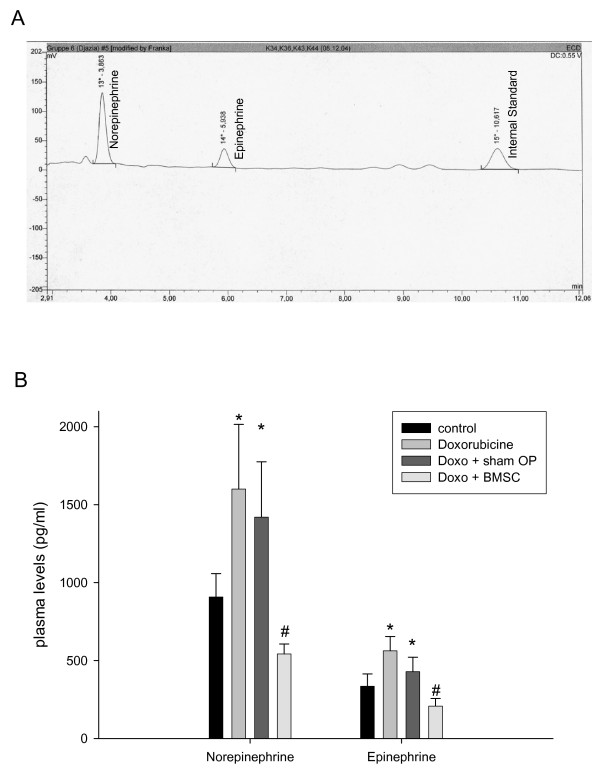
upper panel (A): original plot showing electrochemical detection of norepinephrine, epinephrine and internal standard in rabbit plasma. lower panel (B): Quantitative data for plasma catecholamines in normal rabbits, in rabbits with doxorubicine-induced heart failure, rabbits suffering from heart failure after sham-operation, and BMSC transplanted rabbits with failing hearts, given as MEANS ± SEM of n = 6 rabbits in each group. Significant differences versus normal animals are indicated by a * (p < 0.05), while significant changes versus sham-operated rabbits are indicated by a # (p < 0.05).

Regarding catecholamine plasma concentrations HPLC revealed clear and sharp peaks for norepinephrine, epinephrine and internal standard (fig. 5, panel A). In rabbits with untreated heart failure we found significantly enhanced norepinephrine and epinephrine levels (fig. 5, panel B). The same holds true for rabbits suffering from heart failure receiving sham-operation. In contrast, in rabbits with heart failure treated with BMSC transplantation norepinephrine and epinephrine plasma levels were significantly lower than in untreated or sham-operated rabbits (fig. 5, panel B).

In line with these findings, invasive in-vivo contractility testing with Millar catheter revealed significantly improved contractility in the BMSC treated animals (see table 1). Thus, dP/dtmax and dP/dtmin under stimulation with 1.5 μg/kg dopamine were largely decreased in failing hearts and sham-operated failing hearts, while in failing hearts which received BMSC transplantation this parameter was significantly enhanced (see table 1). Pearson correlation of β-adrenoceptor density and contractility (dP/dt_max_) revealed a significant correlation with R^2 ^= 0.887 and p < 0.05.

**Table 1:** Contractility given as dP/dt_max _and dP/dt_min _(mm Hg/s), body weight (g) and heart weight/body weight ratio (mg/g), given as MEANS ± SEM for each group. Significant differences versus normal animals are indicated by a * (p < 0.05), while significant changes versus sham-operated rabbits are indicated by a # (p < 0.05).

Series	dP/dt_max_	dP/dt_min_	Body weight	Heart weight/body weight
Control	2603 ± 281	-2102 ± 241	3633 ± 83	3.23 ± 0.10
Doxorubicine	1001 ± 126*	-759 ± 86*	2587 ± 79*	4.12 ± 0.27*
Sham-operated	1290 ± 138*	-860 ± 93*	2544 ± 84*	4.40 ± 0.20*
BMSC transplanted	2084 ± 151#	-1616 ± 97#	2586 ± 79*	4.10 ± 0.20*

**Figure 6:**
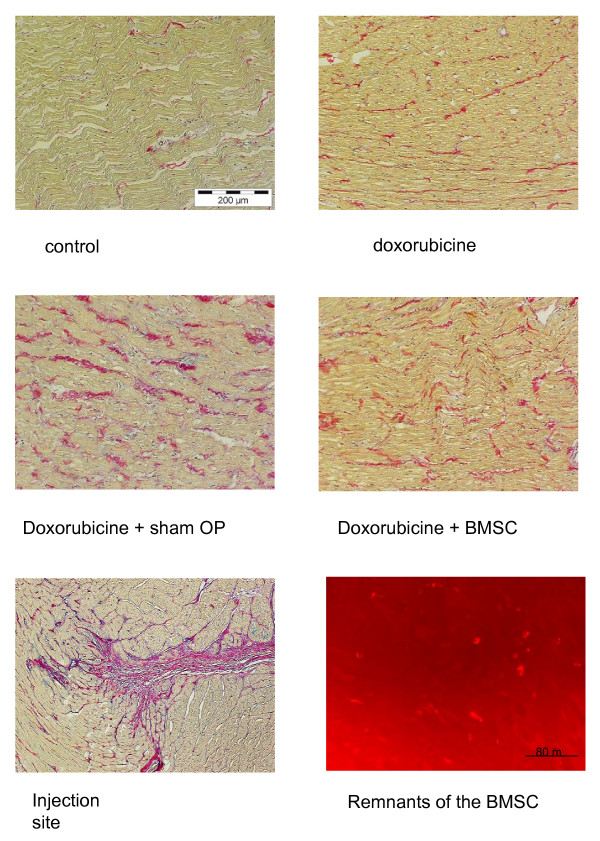
Picrosirius red staining of the hearts of the control, doxorubicine, sham-operated, and BMSC-transplanted group. As can be seen there is a mild interstitial fibrosis in all hearts, which is slightly more pronounced in the failing hearts. At the lower left, an injection site is shown, exhibiting the formation of a scar. At the lower right, red fluorescent cell detritus and some intact fluorescing cells are shown, which were found close to the injection site.

By histological investigation of the hearts we found 2–3 intact cells/visual field (magnification 200 x) and some remnants showing Vybrand DiI red fluorescence in BMSC treated hearts in the vicinity of the injection marks, but not in sham-operated or control hearts. However, these few cells were found only in close neighbourhood of the injection. In that area we also detected red fluorescent cell detritus (fig. 6). We did not observe marked signs of an inflammatory response such as leukocyte or lymphocyte infiltration. However, in most normal, failing and treated hearts a mild interstitial fibrosis was observed (fig. 6), which was slightly more expressed in all failing hearts. At the site of injection, we found a fibrotic scar (fig. 6). In all animals suffering from heart failure body weight was reduced and heart weight/body weight ratio was increased. There were no significant differences among the heart failure groups (table 1).

**Figure 7:**
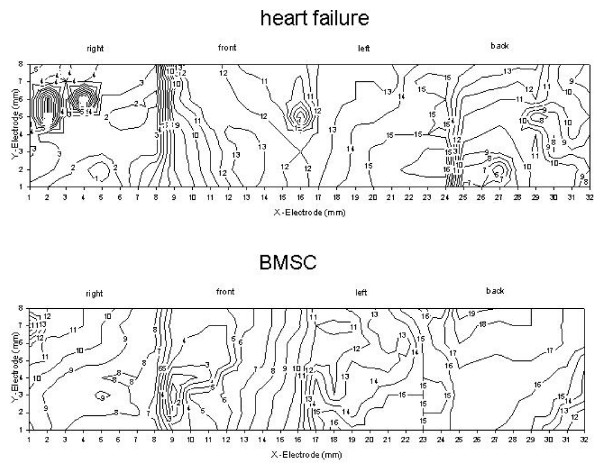
Original isochrones (spacing 1 ms) of the epicardial activation of the hearts surface in an untreated failing heart (upper) and a BMSC-treated (lower) failing heart (Y-electrode row 1 is caudal, while Y-electrode 8 is cranial; X-electrode location is indicated in the plot as right, front, left or back wall of the ventricles). Cumulative data for all hearts of this series are given in table 2.

Regarding the subgroup of hearts which underwent mapping experiments, we found regularly beating hearts with undisturbed propagation of the cardiac activation wave front (fig. 7). The total activation time (TAT) was not different among the groups as was the standard deviation of the activation times at the 256 electrodes, which reflects the inhomogeneity of the activation process (table 2; fig. 7). Thus, inhomogeneity of the activation process was not enhanced by BMSC transplantation. The activation-recovery intervals (ARI) were not changed by BMSC injection versus sham-operated animals and the dispersion of ARI also was not changed (table 2). We never observed spontaneously depolarizing areas in the ventricles or sustained arrhythmia. The only finding was that in all failing heart groups ARI was somewhat prolonged but without reaching the level of significance.

**Table 2:** Electrophysiological findings of healthy, doxorubicine-treated and BMSC-treated rabbits in comparison to sham-operated animals. (Abbreviations: TAT: total activation time; ARI = activation-recovery interval; SD(ACT) : standard deviation of activation time; DISP: dispersion defined as standard deviation of activation-recovery interval). The differences among the groups were not significant.

Series	TAT	SD(ACT)	ARI	DISP
Control	17.3 ± 2.7	0.24 ± 0.04	128.4 ± 16.3	18.4 ± 2.4
Doxorubicine	18.1 ± 3.1	0.26 ± 0.04	147.1 ± 6.6	13.3 ± 1.0
Sham-operated	18.7 ± 4.3	0.28 ± 0.05	143 ± 7.4	14.1 ± 0.8
BMSC transplanted	19.5 ± 1.8	0.28 ± 0.03	141.6 ± 10.3	18.8 ± 3.5

## Discussion

The results of our study clearly show -to the best of our knowledge for the first time- an improvement and -as assessed by β-adrenoceptor density- long-term improvement of cardiac hemodynamic function by autologous mesenchymal bone marrow stem cell transplantation in a model of non-ischemic cardiomyopathy. Pathophysiologically the findings can be explained by the assumption that doxorubicine-induced heart failure results in compensatory catecholamine release (see fig. 5, panel B) which in turn leads to β-adrenoceptor down-regulation (see fig. 4). It is known that changes in β-adrenoceptor density as a consequence of altered catecholamine levels or β-blocker therapy in chronic heart failure mostly need days or weeks to develop being related to the severity of the disease [13,14,25-27,36]. If according to these studies a therapy of heart failure is hemodynamically effective, one has to assume that catecholamine release should diminish and β-adrenoceptor density increased towards normal values. This very point could be demonstrated with the data in this paper showing a normalization of both catecholamine levels and β-adrenoceptor density together with improved contractility after BMSC transplantation. Since it has been shown that normalization of β-adrenoceptor density needs longer time periods with improved hemodynamics [25,26], we assume that these findings indicate a long-term improvement of cardiac function by BMSC transplantation within the limitations of this model. The positive and significant correlation between β-adrenoceptor density and contractility (R^2 ^= 0.887, p < 0.05) supports the hypothesis on a relationship between these two parameters. A point worth discussing is the finding that the effects of BMSC transplantation are more pronounced in the septum than in the free left ventricular wall: the BMSC were injected in the free left ventricular wall at 10 sites. Each injection produced a scar (fig. 6) at the site of injection. In a tiny heart as the rabbit heart 4 injections and the resulting scars will increase the amount of fibrous tissue, which will not occur in the septum, since there are no injections made due to technical reasons of the operation procedure. The scars and the resulting change in the tissue composition in the left free wall may overshadow in parts the effects of BMSC transplantation on β-adrenoceptor density, which can be better seen in the non-injected septum, if the hemodynamic situation is improved.

Regarding the relation between catecholamine levels and β-adrenoceptor density and contractility, it is difficult to say what is first. One may speculate, that the improved hemodynamic situation should result in decreased catecholamine levels, and that this will consequently lead to attenuation of β-adrenoceptor down-regulation. However, it is also possible that BMSC-transplantation primarily leads to enhanced β-adrenoceptor density or attenuated down-regulation possibly by a paracrine effect, which should then -analogous to beta-blocker treatment- lead to an improved hemodynamic situation.

Numerous studies showed positive effects of BMSC transplantation in ischemic heart disease or acute myocardial infarction (e.g. [4,8,11]) either by direct infiltration or by intracoronary stem cell injection (for a recent review see [1]). It has been assumed that ischemia leads to expression of yet unknown homing factors attracting the stem cells [37]. In non-ischemic cardiomyopathy it is unclear whether such homing factors exist and, thus, we decided to use direct myocardial infiltration technique for BMSC application. The finding that we only found BMSC or their remains in close vicinity of the injection speaks somewhat against a predominant role of homing factors in this model of non-ischemic cardiomyopathy although for a final statement on this point (which was not the focus of our study) experiments using e.g. intracoronary application will be needed. Nevertheless, our data present the first evidence for the efficacy of BMSC transplantation in non-ischemic cardiomyopathy.

Regarding the underlying mechanisms of BMSC transplantation there is an ongoing debate discussing factors such as angiogenetic effects, transdifferentiation, cell fusion and the release of paracrine factors [1,38]. First of all, the lack of effect of medium injection (see sham-operated animals) demonstrates that it is not the trauma of operation and injection; moreover, one could hypothesize that the BMSC injection might induce an inflammatory response (although the cells are autologous). However, we have no indication of a inflammatory lymphocyte or leukocyte infiltration of the ventricular myocardium, which clearly is against the hypothesis of an unspecific inflammation. Moreover, the sham operated hearts were also injected with medium, but did neither show inflammatory response nor therapeutic effect. A myocardial transdifferentiation of BMSC, a hypothesis broad forward by e.g. Tomita et al. [4] and others [5,39], cannot be completely ruled out, but with 2–3 cells/visual field at 200 x magnification and only in vicinity of the injection) we have no indication for an amount of cells undergoing transdifferentiation (or even being present as they are) which would be large enough to explain the contractile and hemodynamic improvement. Instead, one could imagine that the injected cells release paracrine factors, yet unknown (or see above), which cause a response of the surrounding myocardial tissue (including all types of cells) being responsible for the process. Future studies will have to be directed to the investigation of this hypothesis and the identification of possible paracrine effects, if existing.

Regarding the safety of BMSC transplantation the results obtained with skeletal myoblasts indicate a possible arrhythmogenic risk of cell therapy [40-42], so that a deeper investigation seems to be necessary. Therefore, we decided to investigate a subset of hearts by means of epicardial potential mapping. We checked the number of epicardial breakthroughpoints, looked for arrhythmogenic foci and signs of inhomogeneity such as delayed activation or increased dispersion. None of these parameters was different in BMSC transplanted hearts from failing hearts without therapy. This does not completely rule out arrhythmogeneic effects of this therapy in other (in particular later) phases of the therapy but these results show that at least after 4 weeks no autonomic areas or non-excitable zones (larger than 0.5 mm^2^) have been induced by the therapy. Taking the low number of surviving cells into account this seems reasonable. Thus, with all caution, we have at present no indication of an arrhythmogenic effect of autologous BMSC transplantation in non-ischemic cardiomyopathy within the limitations of this model.

## Conclusion

The mechanism by which stem cell transplantation leads to improvement of cardiac function is still uncertain and further studies are on the way to elucidate this question. The small number of surviving BMSC in our study is in favour of the assumption of indirect effects such as e.g. paracrine effects. Our data clearly show a long-term improvement in cardiac function by autologous stem cell therapy in non-ischemic cardiomyopathy, so that we conclude that this might be an interesting new therapeutic approach to the treatment of non-ischemic cardiomyopathy.

## Competing interests

The author(s) declare that they have no competing interests.

## Authors' contributions

Stefan Dhein had the idea of the study, performed data analysis, statistics, graphics, and wrote the paper. Jens Garbade did the surgery on the animals, isolated and characterised the stem cells, and performed investigations of contractility; Jan Fritz Gummert and Friedrich-Wilhelm Mohr, both helped with the surgical techniques and added important comments to the paper; Djazia Rouabah, Getu Abraham, and Fritz-Rupert Ungemach performed the radioligand binding studies and analysed the binding curves.

Katja Schneider performed anaesthesia of the animals and the postoperative animal care. Cris Ullmann developed the software for the pressure volume loops analysis and analysed these data, Heike Aupperle performed all histological work on the hearts, and analysed the cell number of surviving stem cells.
